# Cognitive Processing Speed Assessment Combined With Gait Performance Improves Prediction of Shunt Surgery Responsiveness in Idiopathic Normal Pressure Hydrocephalus

**DOI:** 10.1002/cns.71175

**Published:** 2026-09-23

**Authors:** Ziang Geng, Zeyi Yang, Pengtao Li, Xiao Zhang, Yihao Chen, Jianbo Chang, Rui Yin, Sishuai Sun, Xiaoyu Liu, Congcong Deng, Yue Wang, Ziyuan Fang, Chenhui Mao, Jing Gao, Ying Mao, Junji Wei

**Affiliations:** ^1^ Department of Neurosurgery, Peking Union Medical College Hospital, Peking Union Medical College Chinese Academy of Medical Sciences Beijing China; ^2^ Department of Neurology, Peking Union Medical College Hospital, Peking Union Medical College Chinese Academy of Medical Sciences Beijing China; ^3^ Departement of Neurosurgery, Huashan Hospital, Shanghai Medical College Fudan University Shanghai China

**Keywords:** cognitive processing speed assessment, idiopathic normal pressure hydrocephalus, predictive model, symbol digit modalities test, trail making test‐A

## Abstract

**Background:**

Idiopathic normal pressure hydrocephalus (iNPH) is a reversible neurological disorder. Cerebrospinal fluid tap test (CSF TT) is widely used to predict shunt surgery responsiveness, but its predictive performance remains limited, partly because cognitive evaluation has received insufficient emphasis.

**Methods:**

72 patients diagnosed with possible iNPH were enrolled and underwent CSF TT. Gait performance was assessed using the 5‐m timed up and go test (TUG) and the 10‐m walking test. Cognitive processing speed was assessed using the trail making test Part A (TMT‐A) and the Symbol Digit Modalities Test (SDMT). Improvement rates at different time points were analyzed, and a combined predictive model (Combo model) was constructed using firth logistic regression.

**Results:**

Cognitive processing speed progressively declined with increasing disease severity and showed strong correlations with gait abnormalities. Improvement rates at 24 h showed the highest or near‐highest discriminative performance for shunt responsiveness across most measures. Both TMT‐A and SDMT showed significant improvement at 24 h after CSF release. Optimal improvement rate thresholds were 50% for TMT‐A and SDMT and 10% for TUG and 10‐m walking test. The Combo model yielded an apparent AUC of 0.935.

**Conclusion:**

Cognitive processing speed reflects disease severity in patients with iNPH. Short‐term improvement in cognitive processing speed after CSF release suggests potential benefits from shunt surgery. A combined model integrating cognitive processing speed and gait performance measures can substantially enhance the prediction of shunt surgery outcomes.

## Graphical abstract

Overview of the study workflow illustrating baseline assessment of cognitive function and gait performance in patients with iNPH, followed by CSF tap test and post‐tap test reassessment. Functional improvement rates were calculated based on pre‐TT performance, with 24 h selected as the optimal assessment time point. A combined predictive model integrating digital cognitive and gait‐related measures was constructed to estimate shunt surgery responsiveness.

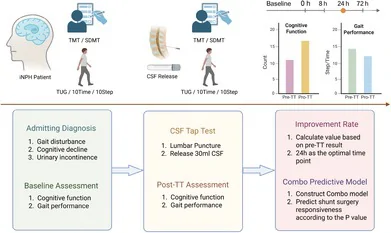

**Abbreviations**
10Step: 10‐m walking step
10Time: 10‐m walking time
AUC: area under curve
CI: confidence interval
CSF: cerebrospinal fluid
DCA: decision curve analysis
FDR: false discovery rate
iNPH: idiopathic normal pressure hydrocephalus
iNPHGS: iNPH grading scale
IQR: inter quartile range
LP Shunt: lumboperitoneal shunt
MMSE: mini mental status exam
ROC: receiver operating characteristic curve
SDMT: symbol‐digit modalities test
TGT: tandem gait test
TMT: trail making test
TT: tap test
TUG: 5‐m timed up and go test
VP Shunt: ventriculoperitoneal shunt

## 1 Introduction

Idiopathic normal pressure hydrocephalus (iNPH) is a neurological condition characterized by ventricular enlargement and abnormal cerebrospinal fluid (CSF) circulation. Patients typically present with the classic triad of gait disturbance, cognitive decline, and urinary incontinence. The underlying pathophysiology is closely linked to disrupted CSF dynamics and altered brain tissue compliance [1]. CSF shunt surgery remains the mainstay of treatment for alleviating symptoms and improving daily functioning in affected individuals [2]. In current clinical practice, the CSF tap test (TT) serves as a key tool for predicting surgical outcomes. This test involves draining 30 mL of CSF via lumbar puncture, with symptom assessment performed both before and after the procedure. Marked symptom improvement following the tap test generally indicates a favorable response to subsequent shunt placement [3]. However, the degree of improvement observed after the tap test does not always reliably predict shunt effectiveness. Several factors may account for this discrepancy, including variations in the timing of assessments and differences in evaluation methods.

Beyond gait impairment, cognitive decline represents another core feature of iNPH. Recent studies have revealed that cognitive deficits in these patients extend beyond global intellectual deterioration, with particularly prominent impairment in processing speed and attention‐related functions. These cognitive domains encompass sustained attention, information processing speed, symbol‐digit association, and executive task performance, all of which are closely tied to frontal lobe function and subcortical circuit integrity [4]. Such deficits often manifest as reduced efficiency in numerical processing, symbol substitution, and multitasking, significantly compromising patients' ability to manage daily activities and complete complex tasks independently [5]. Nonetheless, conventional CSF tap test assessments have largely focused on changes in gait and motor function, with insufficient attention given to processing speed and attention‐related domains. As a result, potential cognitive benefits in some patients may go unrecognized. The trail making test (TMT) and the symbol digit modalities test (SDMT) are two well‐established tools for quantifying these cognitive functions in clinical settings. The TMT, available in Part A and Part B versions, primarily evaluates task‐switching ability and executive control involving numerical sequences, whereas the SDMT assesses information processing speed and symbol‐digit pairing efficiency [6]. In the present study, these domains are collectively referred to as cognitive processing speed assessment.

Accordingly, this study employed TMT‐A and SDMT as the primary tools for evaluating cognitive processing speed. We systematically examined the relationship between these cognitive measures and disease severity in iNPH patients, aiming to elucidate the role of such cognitive impairment in the pathophysiology of iNPH and its potential value as a clinical indicator. We further analyzed the predictive performance of improvement rates across various outcome measures at different time points following the CSF tap test to explore the timing of functional assessment after the procedure. Building on these findings, we compared changes in processing speed and attention‐related cognitive performance at the optimal assessment time point. We then integrated TMT‐A and SDMT results with gait and mobility measures, specifically the 5‐m timed up and go test (TUG) and the 10‐m walk test, to develop a comprehensive predictive model (referred to as the Combo model) for estimating shunt surgery outcomes. By integrating cognitive processing speed and gait performance, this approach aims to improve the estimation of shunt responsiveness and provide a more objective, quantifiable framework to guide preoperative patient selection and individualized treatment planning.

## 2 Methods

### 2.1 Patient Enrollment and Clinical Evaluation

This study included patients with possible idiopathic normal pressure hydrocephalus (iNPH) who were consecutively admitted to the Department of Neurosurgery at Peking Union Medical College Hospital between January 2023 and September 2025. The inclusion criteria were as follows: (1) diagnosis of possible iNPH according to the international guidelines and the Chinese expert consensus [7]; (2) successful completion of the cerebrospinal fluid tap test (CSF TT); and (3) completion of gait and cognitive processing speed assessments at designated time points before and after the CSF TT. The exclusion criteria were as follows: (1) secondary causes of hydrocephalus; (2) failure to undergo the CSF TT or intolerance to the removal of 30 mL of cerebrospinal fluid; and (3) incomplete functional assessments before or after the CSF TT. Based on these criteria, a total of 72 patients were enrolled. Patients were considered for shunt surgery based on the overall clinical evaluation after the CSF TT in accordance with routine clinical practice at our institution. Consequently, post‐operative outcome data were available only for patients who underwent shunt surgery. The study was approved by the Ethics Committee of Peking Union Medical College Hospital (2024‐I2M‐CT‐B‐022), and written informed consent was obtained from all patients or their legal representatives. Additionally, all patients completed the Mini‐Mental State Examination (MMSE) and the Idiopathic Normal Pressure Hydrocephalus Grading Scale (iNPHGS) upon admission.

### 2.2 CSF Tap Test and Assessment

For the CSF TT, 30 mL of cerebrospinal fluid was removed via lumbar puncture. Gait performance and cognitive processing speed were assessed before the procedure and at predefined time points after the CSF TT. Based on the timing of hospital admission, the 72 enrolled patients were divided into two cohorts. Cohort 1 consisted of 28 patients admitted between January 2023 and February 2024, who underwent functional assessments at 8, 24, and 72 h after the CSF TT. Cohort 2 included 44 patients admitted between March 2024 and September 2025, who were assessed at 24 and 48 h after the CSF TT.

### 2.3 Gait Performance Assessment

Gait performance was evaluated using the TUG test and the 10‐m walk test. Each patient completed these assessments before the CSF TT and at the predefined post‐CSF TT assessment time points. The 10‐m walk test yielded two outcome measures: 10‐m walk time (10Time) and 10‐m step count (10Step). For the TUG test, participants were instructed to rise from a seated position, walk 5 m, turn around, return to the chair, and sit down again. The total time required to complete the task was recorded to assess gait initiation, turning ability, and overall mobility. For the 10‐m walk test, participants walked 10 m at their usual pace. The time required to complete the walk (10Time) and the number of steps taken (10Step) were recorded to evaluate walking speed and gait characteristics. Each assessment was performed twice, and the average of the two trials was used for analysis. All assessments were video‐recorded. Using pre‐TT results as the baseline reference, improvement rates were calculated for each measure at every post‐TT point. The formula for calculating the improvement rate (*dX*) in gait and motor function is as follows: $\mathrm{dX}=\frac{X_{\mathrm{pre}}-X_{\mathrm{pro}}}{X_{\mathrm{pre}}}$.

### 2.4 Cognitive Processing Speed Assessment

Cognitive processing speed was assessed using the TMT‐A and SDMT. These standardized paper‐and‐pencil tests were selected because they provide brief and practical measures of cognitive processing speed that can be readily incorporated into routine clinical assessment. Each patient completed both tests before the CSF TT and at the predefined post‐CSF TT assessment time points. For the TMT‐A, participants were required to connect a series of numbered circles in sequential order as quickly as possible. The number of correct connections completed within the specified time limit was recorded as a measure of visual scanning ability and cognitive processing speed. For the SDMT, participants were provided with a reference key pairing symbols with corresponding numbers and were instructed to substitute each symbol with its matching digit as quickly as possible within the specified time limit. The number of correct responses was recorded as a measure of information processing speed and sustained attention. Using pre‐TT results as the baseline reference, improvement rates were calculated for each measure at every post‐TT point. The formula for calculating the improvement rate (*dZ*) in cognitive processing speed is as follows: $\mathrm{dZ}=\frac{Z_{\mathrm{pro}}-Z_{\mathrm{pre}}}{Z_{\mathrm{pre}}}$.

### 2.5 CSF Shunt Surgery

A total of 51 patients underwent ventriculoperitoneal (VP) or lumboperitoneal (LP) shunt surgery following the CSF TT. Postoperative outcomes were assessed at the 3‐month follow‐up using the iNPHGS. Patients with an improvement of more than one point in the total iNPHGS score compared with baseline were classified as responders, whereas those with an improvement of one point or less were classified as non‐responders. Postoperative assessments were performed by outpatient neurosurgeons during routine follow‐up. Assessors were not blinded to previous clinical evaluations.

### 2.6 Statistical Analysis

Continuous variables were presented as medians with interquartile ranges (IQRs), whereas categorical variables were expressed as counts and percentages. Spearman's rank correlation coefficient was used to evaluate correlations between continuous variables. The Kruskal‐Wallis test was applied for comparisons involving ordinal variables, and the Wilcoxon signed‐rank test was used to compare paired continuous variables. Pearson's chi‐squared test was performed to analyze differences in categorical variables. All analyses were conducted using available‐case data, and no imputation for missing values was performed.

The diagnostic performance of individual indicators was evaluated using receiver operating characteristic (ROC) curve analysis. For each indicator, the Youden‐derived probability threshold was determined by maximizing the Youden index (*J* = sensitivity + specificity − 1). The area under the ROC curve (AUC) and its 95% confidence interval (CI) were estimated using the DeLong method. Sensitivity and specificity at the Youden‐derived threshold were reported together with their exact (Clopper‐Pearson) 95% confidence intervals.

An integrated prediction model for shunt responsiveness was developed using Firth penalized logistic regression to reduce small‐sample bias and mitigate potential separation resulting from the limited number of non‐responders. Model discrimination was evaluated using ROC curve analysis, whereas model calibration and clinical utility were assessed using calibration curves and decision curve analysis (DCA), respectively. Internal validation of the integrated prediction model was performed using 2000 bootstrap resamples. The predictor cutoff values derived from the original surgical cohort were fixed during bootstrap validation and were not re‐estimated within each bootstrap resample. Thus, the bootstrap procedure evaluated model optimism conditional on the originally selected cutoff values rather than reproducing the entire cutoff‐selection process. Apparent model performance, optimism, and optimism‐corrected performance were estimated. The optimism‐corrected AUC and Brier score were calculated. Bootstrap optimism‐corrected calibration curves and decision curve analysis were used to evaluate model calibration and clinical utility. Bootstrap optimism‐corrected calibration curves and decision curve analysis were used to evaluate model calibration and clinical utility. Differences in AUCs among prediction models were assessed using the paired DeLong test. To account for multiple comparisons, *p* values were adjusted using the Benjamini‐Hochberg procedure to control the false discovery rate (FDR), and FDR‐adjusted *p* values < 0.05 were considered statistically significant. All statistical analyses and figure visualizations were performed using Stata version 13.0 (StataCorp LLC, College Station, TX, USA) and R version 4.4.1 (R Foundation for Statistical Computing, Vienna, Austria). All statistical tests were two‐sided, and *p* < 0.05 was considered statistically significant unless otherwise specified.

## 3 Results

### 3.1 Baseline Clinical Characteristics of Patients With iNPH

A total of 72 patients with possible iNPH were ultimately enrolled in this study. Cohort 1 (assessed at 8 h/24 h/72 h) included 28 patients, while Cohort 2 (assessed at 24 h/48 h) included 44 patients. The median age was 73 years. Males outnumbered females, with 53 men (74%) and 19 women (26%). The median MMSE score was 23, and the median iNPHGS score was 4. The median Evans Index was 0.353 (0.318, 0.381), and DESH was present in 37 patients (51%). Among the 72 patients, 42 (58%) demonstrated a positive Romberg sign, and 50 (69%) exhibited a positive tandem gait test (TGT). For processing speed and attention‐related cognitive measures, the median TMT‐A score was 15, and the median SDMT score was 10. For gait and motor function, the median TUG time was 25 s, the median 10Time was 17 s, and the median 10Step count was 27 steps.

Most baseline demographic, imaging, and functional characteristics were similar between the two cohorts. However, Cohort 1 had a higher median MMSE score than Cohort 2 and a higher proportion of patients with a positive Romberg sign. Notably, no significant between‐cohort differences were observed in the baseline functional measures subsequently used in the timing analysis, including TMT‐A, SDMT, TUG, 10Time, and 10Step (Table 1).

**TABLE 1:** Baseline demographic, clinical, and imaging characteristics of iNPH patients.

Characteristics	Overall	Cohort 1 (8 h/24 h/72 h)	Cohort 2 (24 h/48 h)	Exact *p* values
	*N* = 72	*N* = 28	*N* = 44	
Age	73 (69, 76)	73 (68, 77)	72 (69, 75)	0.677
Gender				
Male	53 (74%)	21 (75%)	32 (73%)	1.000
Female	19 (26%)	7 (25%)	12 (27%)	
MMSE	23 (17, 26)	25 (21, 28)	21 (15, 24)	0.026
iNPHGS	4 (3, 6)	5 (3, 6)	4 (3, 6)	0.221
Romberg				
Positive	42 (58%)	22 (79%)	20 (45%)	0.007
Negative	30 (42%)	6 (21%)	24 (55%)	
TGT				
Positive	50 (69%)	23 (82%)	27 (61%)	0.072
Negative	22 (31%)	5 (18%)	17 (39%)	
TMT‐A	15 (8, 24)	18 (10, 25)	15 (6, 22)	0.113
SDMT	10 (3, 17)	11 (5, 20)	10 (3, 15)	0.614
TUG	25 (18, 36)	23 (17, 37)	26 (18, 34)	0.926
10Time	17 (14, 24)	16 (12, 21)	18 (14, 26)	0.138
10Step	27 (23, 29)	27 (20, 35)	28 (23, 42)	0.270
Evan index	0.353 (0.318, 0.381)	0.362 (0.331, 0.398)	0.350 (0.314, 0.373)	0.162
DESH sign				
Positive	37 (51%)	12 (43%)	25 (57%)	0.334
Negative	35 (49%)	16 (57%)	19 (43%)	

### 3.2 Cognitive Processing Speed Declines With Increasing Disease Severity in iNPH

**FIGURE 1:**
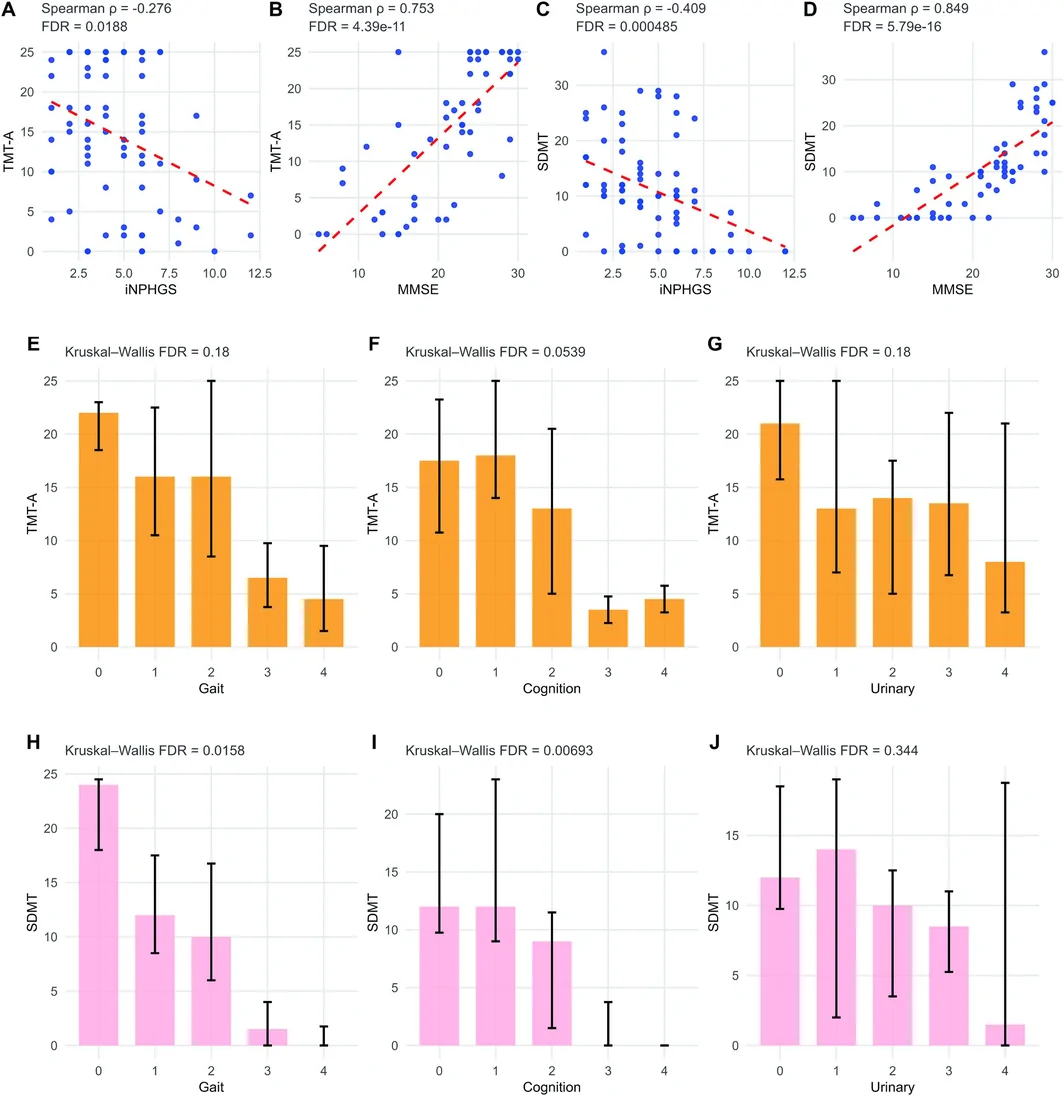
Correlations between digital cognitive performance and clinical disease severity in patients with possible iNPH (*n* = 72). (A–D) Correlations between digital cognitive performance and clinical disease severity, including MMSE scores and iNPHGS scores: (A, B) TMT‐A performance and (C, D) SDMT performance. (E–G) Correlations between TMT‐A performance and the three cardinal symptoms of iNPH (gait disturbance, cognitive impairment, and urinary incontinence). (H–J) Correlations between SDMT performance and the three cardinal symptoms of iNPH.

Correlation analysis was conducted to examine the relationship between cognitive processing speed and disease severity in iNPH patients. The results demonstrated that as hydrocephalus severity increased, reflected by declining MMSE scores and rising iNPHGS scores, performance on TMT‐A and SDMT progressively deteriorated in terms of both accuracy and task completion (Figure 1A–D). This pattern suggests that cognitive processing speed in these domains worsens in parallel with disease progression. Further correlation analysis between TMT‐A, SDMT, and the three cardinal symptoms of iNPH (gait disturbance, cognitive impairment, and urinary incontinence) revealed that these two tests not only capture deficits in processing speed and attention but also show some association with gait abnormalities. This finding pointed to a dual sensitivity of these measures to both cognitive and motor dimensions (Figure 1E–J). Although TMT‐A and SDMT primarily assess information processing speed and executive performance involving numerical and symbolic tasks, their correlation with gait impairment further underscores a close interplay between cognitive dysfunction and motor deficits in iNPH.

### 3.3 Exploratory Comparison of Predictive Performance Across Post‐CSF TT Assessment Time Points

**FIGURE 2:**
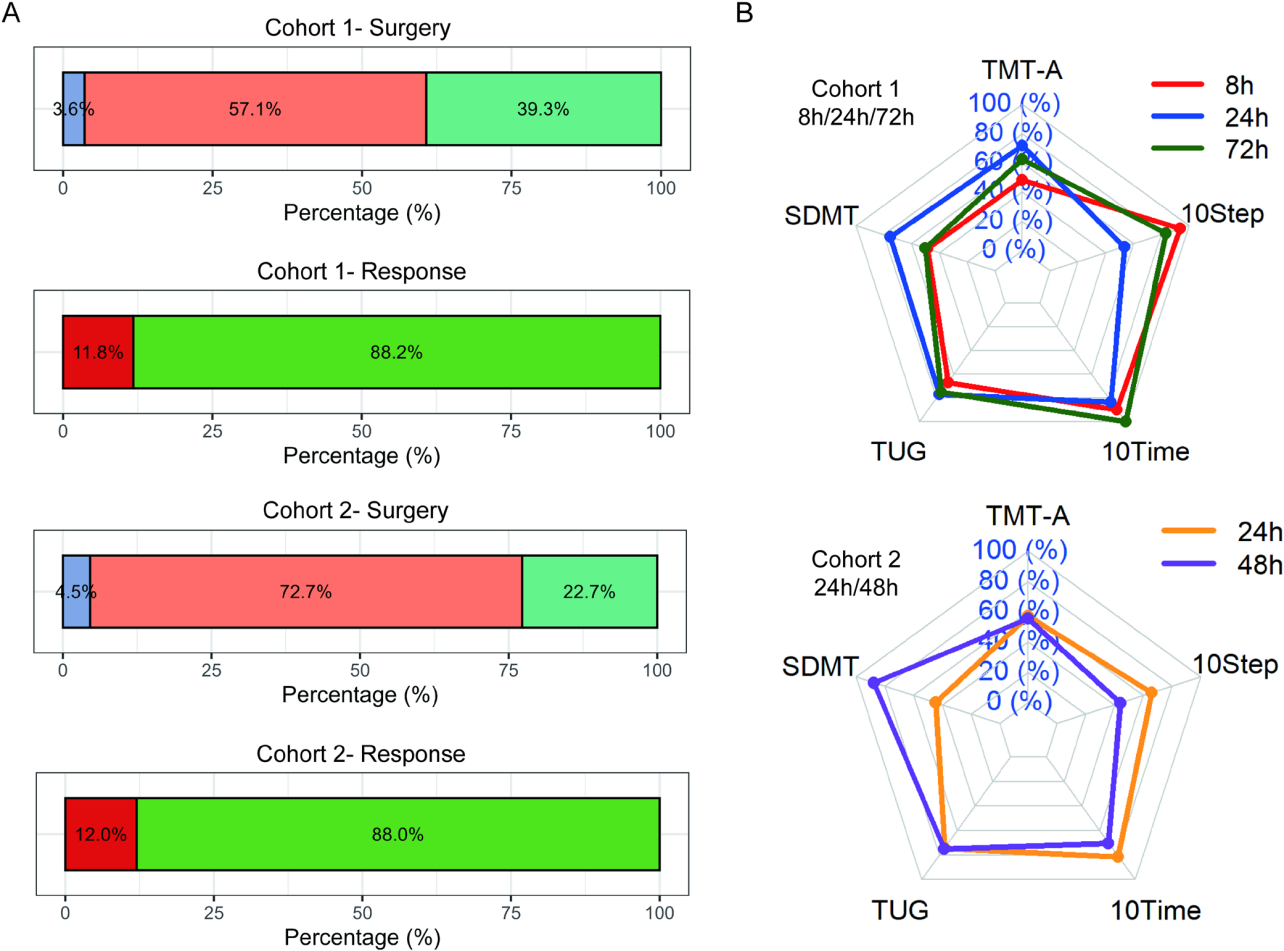
Surgical outcomes and time‐dependent predictive performance of functional improvement after CSF tap test. (A) Surgical treatment and postoperative outcomes in the overall study cohort (*n* = 72), including 28 patients in Cohort 1 and 44 patients in Cohort 2. (B) Comparison of the predictive performance of functional improvement rates at 8 h/24 h/72 h in Cohort 1 (*n* = 28) and 24 h/48 h in Cohort 2 (*n* = 44) after CSF TT.

Among the 28 patients in Cohort 1, 17 ultimately underwent shunt surgery. The vast majority received ventriculoperitoneal (VP) shunt surgery, with only one patient undergoing lumboperitoneal (LP) shunt surgery. The remaining 40% of patients did not proceed to surgical intervention. Of those who underwent surgery, 88.2% were classified as responders postoperatively, while 11.7% were deemed non‐responders. The surgical profile in Cohort 2 was similar to that of Cohort 1. Of the 44 patients, 34 received shunt surgery, again predominantly VP shunt surgery, with only 2 patients undergoing LP shunt surgery. The remaining 8 patients did not receive surgery. Among those who underwent the procedure, the postoperative response rate was 88.0%, with a non‐response rate of 12.0% (Figure 2A). Predictive performance for shunt surgery outcomes was evaluated by calculating the area under the curve (AUC) based on improvement rates at each time point. The results indicated that across both Cohort 1 (8 h/24 h/72 h) and Cohort 2 (24 h/48 h), the majority of gait and cognitive measures achieved the highest or near‐highest AUC values at the 24 h assessment. Assessments conducted too early (8 h) or later (48 h, 72 h) demonstrated comparatively lower discriminative accuracy (Figure 2B). Based on this exploratory comparison, the 24 h‐assessment was selected for subsequent analyses because it showed the highest or near‐highest predictive performance across most measures. This selection was data‐driven rather than prespecified and should therefore be interpreted as exploratory.

### 3.4 CSF Drainage Improves Cognitive Processing Speed in iNPH Patients

**FIGURE 3:**
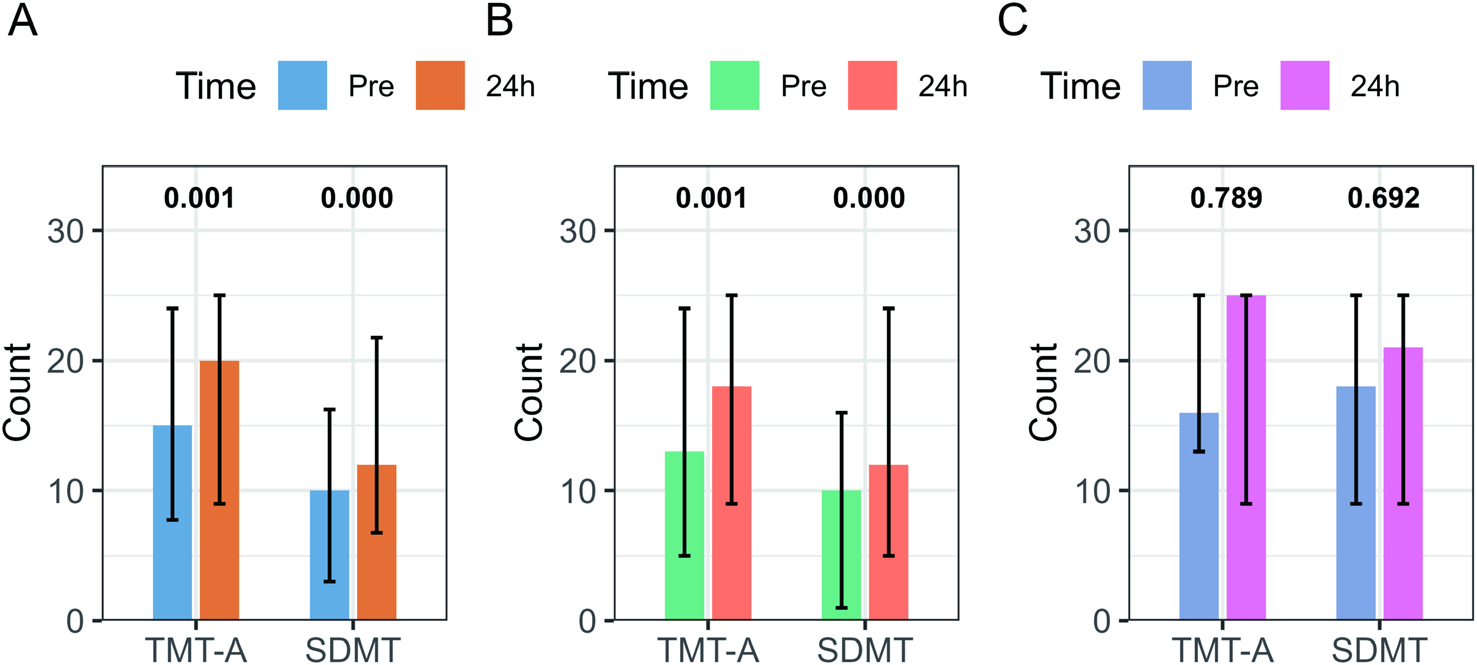
Changes in digital cognitive performance before and 24 h after CSF tap test. (A) TMT‐A and SDMT before CSF TT and at 24 h post‐TT in all enrolled patients (*n* = 72). (B) Subgroup analysis of patients who showed a favorable response to shunt surgery. These patients exhibited marked improvement in TMT‐A and SDMT performance at 24 h after CSF TT (*n* = 51). (C) Subgroup analysis of patients who did not benefit from shunt surgery. In this group, TMT‐A and SDMT scores showed minimal or no improvement after CSF TT (*n* = 21).

To further investigate the immediate effects of CSF release on cognitive processing speed, this study compared TMT‐A and SDMT accuracy before CSF TT and at 24 h post‐TT. The results showed that both test scores significantly improved at 24 h compared with pre‐CSF TT performance (Figure 3A). The improvement in TMT‐A performance suggests recovery in attentional shifting, executive functioning, and cognitive flexibility related to numerical processing. Meanwhile, the increase in SDMT scores reflects enhanced information processing speed and sustained attention. This short‐term improvement following CSF releases indicated that restoration of CSF dynamics may partially alleviate subcortical network dysfunction caused by ventricular enlargement and CSF retention, thereby contributing to cognitive enhancement. Stratified analyses further demonstrated that patients who ultimately benefited from shunt surgery exhibited more pronounced improvements in digital cognitive performance before and after CSF release (Figure 3B). In contrast, patients who did not respond to shunt surgery showed no significant improvement in digital cognitive function after CSF TT (Figure 3C). These findings suggested that TMT‐A and SDMT serve not only as sensitive tools for quantifying cognitive processing speed impairment in iNPH but also as objective indicators for dynamically monitoring CSF TT responsiveness, thereby providing valuable information for predicting surgical outcomes and guiding preoperative patient selection.

### 3.5 Diagnostic Performance of Individual Predictors

**FIGURE 4:**
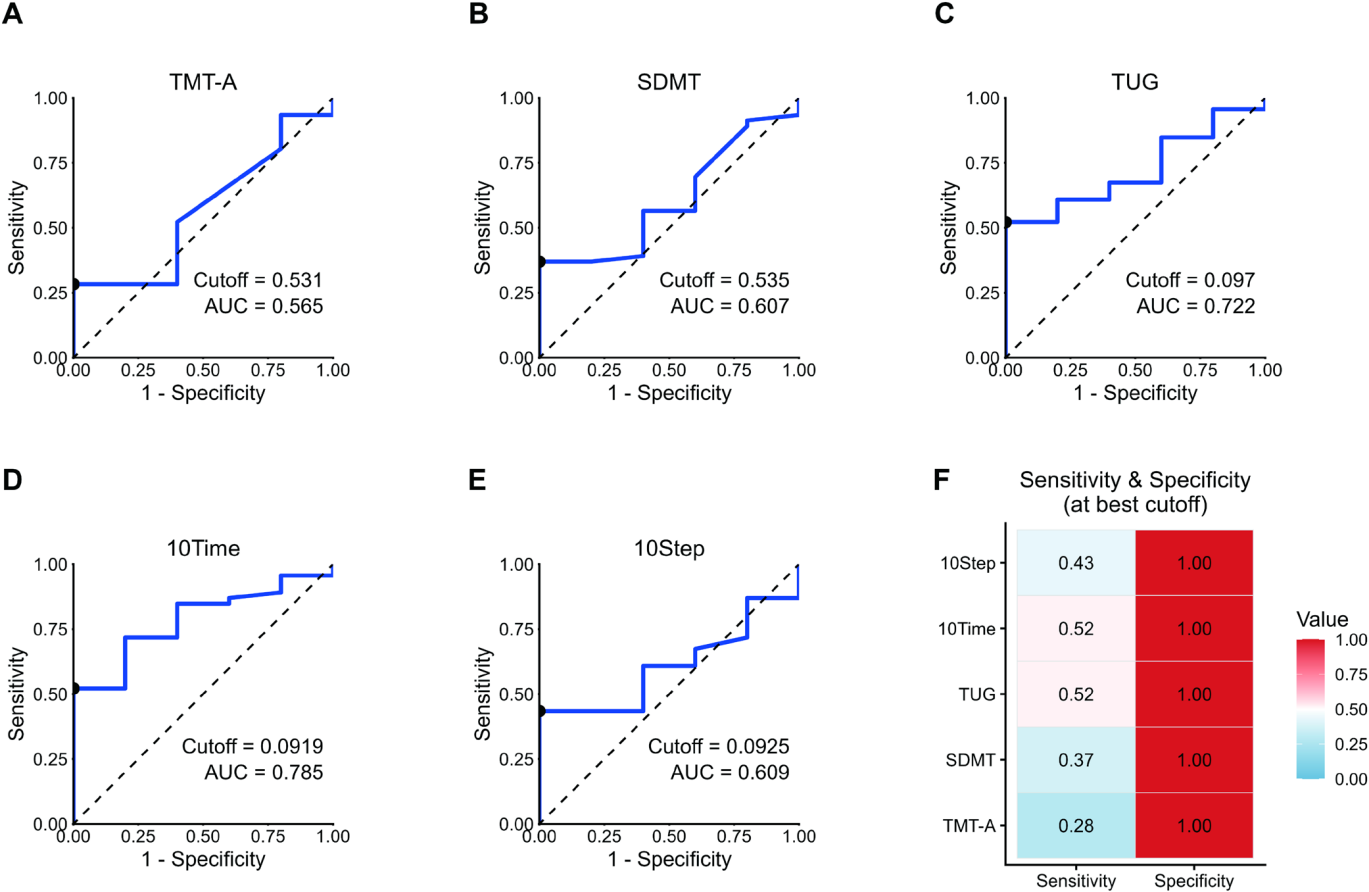
Predictive performance of individual digital cognitive and gait indicators for shunt surgery outcomes in patients with iNPH (*n* = 51). (A–E) ROC curves showing the discriminative performance of individual indicators, TMT‐A improvement rate (A), SDMT improvement rate (B), TUG improvement rate (C), 10Time improvement rate (D), and 10Step improvement rate (E) for identifying shunt‐responsive patients. (F) Summary of the sensitivity and specificity of each indicator at its optimal improvement cutoff value.

To evaluate the predictive value of individual indicators for shunt responsiveness, ROC curve analysis was performed for the 24 h post‐TT improvement rates of TMT‐A, SDMT, TUG, 10Time, and 10Step. Because these variables were continuous, exploratory ROC‐derived probability thresholds were identified by maximizing the Youden index. Patients were subsequently classified as test‐positive or test‐negative according to the direction of association and the corresponding threshold. The exploratory ROC‐derived thresholds were 0.531 for TMT‐A, 0.535 for SDMT, 0.097 for TUG, 0.0919 for 10Time, and 0.0925 for 10Step. The corresponding AUCs were 0.565 (95% CI, 0.302–0.828), 0.607 (95% CI, 0.368–0.845), 0.722 (95% CI, 0.538–0.906), 0.785 (95% CI, 0.612–0.957), and 0.609 (95% CI, 0.412–0.805), respectively (Figure 4A–E). Detailed diagnostic performance, including the exploratory thresholds, sensitivities, specificities, and corresponding 95% confidence intervals, is summarized in Table 2.

**TABLE 2:** Diagnostic performance of individual predictors for shunt responsiveness.

Predictor	Cutoff	AUC (95% CI)	Sensitivity (95% CI)	Specificity (95% CI)
TMT‐A	0.531	0.565 (0.302–0.828)	28.3% (16.0%–43.5%)	100.0% (47.8%–100.0%)
SDMT	0.535	0.607 (0.368–0.845)	37.0% (23.2%–52.5%)	100.0% (47.8%–100.0%)
TUG	0.097	0.722 (0.538–0.906)	52.2% (36.9%–67.1%)	100.0% (47.8%–100.0%)
10Time	0.0919	0.785 (0.612–0.957)	52.2% (36.9%–67.1%)	100.0% (47.8%–100.0%)
10Step	0.0925	0.609 (0.412–0.805)	43.5% (28.9%–58.9%)	100.0% (47.8%–100.0%)

Overall, the individual indicators demonstrated high specificity (100.0%) but relatively low sensitivity (28.3%–52.2%) for predicting shunt responsiveness. Among the five indicators, 10Time exhibited the highest discriminative ability (AUC = 0.785), whereas the cognitive measures (TMT‐A and SDMT) showed comparatively limited standalone predictive performance (Figure 4F). These findings suggested that although individual indicators may help identify patients likely to benefit from shunt surgery, their overall discriminative ability remains modest, supporting the development of an integrated prediction model.

### 3.6 Development and Internal Validation of the Integrated Prediction Model

Given the limited sensitivity of the individual predictors, all five indicators were integrated to develop a comprehensive prediction model (Combo model) incorporating improvements in both cognitive processing speed and gait performance. The model was constructed using Firth penalized logistic regression, with each predictor dichotomized according to its corresponding cutoff value determined by maximizing the Youden index. Each indicator was converted into a binary variable based on its optimal cutoff, and the Combo model was formulated according to the regression equation: $\ln\frac{P}{1-P}=\sum k_{i}\times \beta_{i}+\beta_{0}$. The resulting equation was: $\begin{array}{c} \ln\frac{P}{1\;-\;P}\;=0.3142+\;\mathrm{TMT}\left(A\right)\;\times \;1.5644\;+\;\text{SDMT}\;\times \;2.0331\;+\;\mathrm{TUG}\;\times \end{array}$ $\begin{array}{c} 1.8558\;+\;\text{Time}10\times 0.6377\;+\;\text{Step}10\;\times \;0.8293 \end{array}$. Each variable was assigned a value of 0 or 1, and substitution into the model yielded the predicted probability *p* of shunt surgery effectiveness.

**FIGURE 5:**
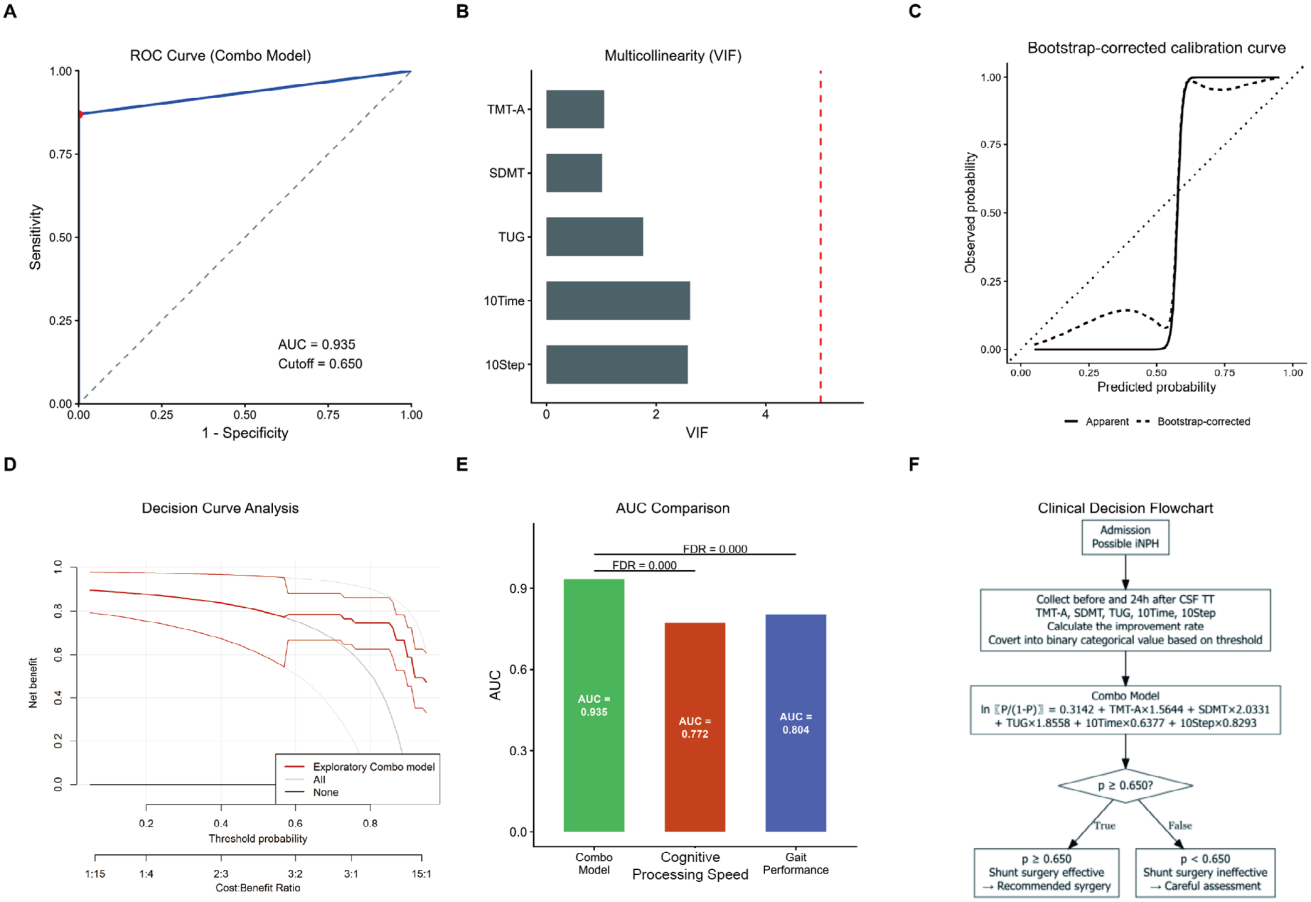
Performance and internal validation of the combined predictive model (Combo model) for shunt surgery responsiveness in iNPH (*n* = 51). (A) Receiver operating characteristic (ROC) curve of the Combo model integrating the 24 h improvement rates of TMT‐A, SDMT, TUG, 10Time, and 10Step. (B) Variance inflation factor (VIF) analysis demonstrating low multicollinearity among the five predictors. (C) Bootstrap‐corrected calibration curve evaluating the calibration performance of the Combo model. (D) Decision curve analysis (DCA) showing the clinical net benefit of the Combo model across different threshold probabilities. (E) Comparison of the area under the ROC curve (AUC) between the Combo model and models based on cognitive processing speed or gait performance alone. (F) Clinical decision flowchart illustrating the application of the Combo model for predicting shunt surgery responsiveness.

The Combo model yielded an apparent AUC of 0.935 (95% CI, 0.886–0.984). The cutoff value was 0.650, corresponding to a sensitivity of 87.0% (95% CI, 73.7%–95.1%) and a specificity of 100.0% (95% CI, 47.8%–100.0%). Compared with the Cognitive Model and the Gait Model, the Combo model achieved the highest diagnostic performance. The diagnostic performance of the three prediction models is summarized in Table 3, and their ROC curves and pairwise comparisons are shown in Figure 5A,E, respectively. Variance inflation factors for the five predictors ranged from 1 to 3, indicating minimal multicollinearity among the variables (Figure 5B). Internal validation was performed using 2000 bootstrap resamples, of which 1986 were successfully completed. The mean optimism in AUC was 0.015, yielding an optimism‐corrected AUC of 0.920. The apparent and optimism‐corrected Brier scores were 0.060 and 0.075, respectively. The bootstrap‐corrected calibration curve demonstrated good agreement between predicted and observed probabilities (Figure 5C). Bootstrap‐corrected decision curve analysis further showed that the Combo model consistently provided greater net clinical benefit than the default treat‐all and treat‐none strategies across a broad range of threshold probabilities (Figure 5D). Based on the cutoff value of 0.650, patients with a predicted probability exceeding this threshold were classified as likely to benefit from shunt surgery (Figure 5F).

**TABLE 3:** Diagnostic performance of prediction models for shunt responsiveness.

Model	Cutoff	AUC (95% CI)	Sensitivity (95% CI)	Specificity (95% CI)
Combo model	0.650	0.935 (0.886–0.984)	87.0% (73.7%–95.1%)	100.0% (47.8%–100.0%)
Cognitive model	0.871	0.772 (0.699–0.845)	54.3% (39.0%–69.1%)	100.0% (47.8%–100.0%)
Gait model	0.813	0.804 (0.733–0.876)	60.9% (45.4%–74.9%)	100.0% (47.8%–100.0%)

## 4 Discussion

The present study provides three principal findings regarding the assessment of patients with possible idiopathic normal pressure hydrocephalus (iNPH). First, impairment in cognitive processing speed was closely associated with overall disease severity and gait dysfunction, supporting the concept that cognitive and motor deficits in iNPH arise from shared pathophysiological mechanisms. Second, functional changes assessed 24 h after the cerebrospinal fluid tap test (CSF TT) provided the most informative indication of short‐term reversibility among the evaluated time points. Third, integrating changes in cognitive processing speed and gait performance improved the estimation of shunt responsiveness compared with individual measures alone. Collectively, these findings suggest that cognitive processing speed represents a clinically meaningful component of the multidimensional manifestations of iNPH and may complement conventional gait assessment during preoperative evaluation.

The results of this study demonstrated that declines in TMT‐A and SDMT accuracy were closely associated with higher MMSE and iNPHGS scores, revealing a pattern in which cognitive processing speed impairment worsens as the disease progresses. This finding is consistent with previous research describing a cognitive‐motor network dysfunction in iNPH, in which both executive cognitive processes and gait regulation rely on the integrity of the frontal‐striatal‐cerebellar network [8]. Ventricular enlargement and CSF‐related alterations in subcortical circuits may produce mechanical traction or perfusion disturbances that affect these interconnected structures simultaneously [9]. Consequently, deterioration in cognitive processing speed may reflect impairment of the broader frontal cognitive‐motor network rather than isolated cognitive decline. This shared neurobiological basis may explain the significant association observed between cognitive processing speed and gait performance in the present study, suggesting that processing speed assessment provides complementary information regarding functional impairment in patients with iNPH.

An important finding of the present study is that cognitive processing speed assessment provides clinically relevant information beyond conventional global cognitive screening. TMT‐A and SDMT were selected because they are brief, paper‐and‐pencil tests that primarily evaluate processing speed, visual attention, and visuomotor performance‐functions that are particularly vulnerable to disruption of frontal‐subcortical circuits in iNPH. Unlike comprehensive neuropsychological batteries, these assessments can be completed within routine outpatient evaluation without specialized equipment, together with standard gait testing, in approximately 15–20 min. The objective of the present study was therefore not to establish the most comprehensive cognitive assessment strategy, but rather to determine whether practical cognitive measures could improve the preoperative evaluation of shunt responsiveness. Nevertheless, cognitive dysfunction in iNPH is multidimensional, and additional domains such as executive function, memory, and global cognition may also contribute to postoperative outcomes. Future studies should investigate whether incorporating broader neuropsychological assessments, including global cognitive screening (e.g., MMSE or MoCA), executive‐function measures such as TMT‐B, and memory assessments, provides incremental predictive value while maintaining clinical feasibility.

The exploratory comparison of multiple post‐CSF TT assessment time points showed that evaluation at 24 h provided the most informative indication of functional reversibility in the present cohort. This observation is consistent with previous studies suggesting that improvement after CSF drainage often evolves over several hours rather than occurring immediately after lumbar puncture [10]. Measurements obtained at earlier time points may underestimate the physiological response to transient normalization of CSF dynamics, whereas later assessments may be influenced by greater interindividual variability. Importantly, both gait performance and cognitive processing speed improved most consistently at 24 h among patients who subsequently achieved a favorable postoperative outcome, supporting the concept that early multidomain functional recovery may reflect preserved neural plasticity and reversibility of frontal‐subcortical network dysfunction [11]. However, the 24 h‐time point was selected exploratorily based on its observed predictive performance across the assessed time points rather than being prespecified as the primary assessment time point. Although the two cohorts differed significantly in baseline MMSE scores and Romberg findings, the functional measures directly used in the timing analysis, including TMT‐A, SDMT, TUG, 10Time, and 10Step, did not differ significantly between cohorts at baseline. Romberg testing was not included in the timing analysis or prediction model. Nevertheless, the difference in MMSE indicates some baseline heterogeneity in global cognitive status between the cohorts, and its potential influence on the comparison of predictive performance across assessment time points cannot be excluded. Accordingly, the timing findings should be interpreted as exploratory and should not be considered as establishing 24 h as the definitive optimal assessment time point. Prospective validation using a standardized assessment schedule in an independent cohort is required.

Previous studies have proposed several strategies for predicting shunt responsiveness in iNPH. Sundström et al. demonstrated that improvement in the Timed Up and Go test provides useful prognostic information, although gait assessment alone cannot fully capture the multidimensional manifestations of the disease [12]. Gallagher et al. subsequently showed that clinically important functional changes after the CSF TT improve prediction of postoperative outcomes [13]. More recently, Cai et al. reported promising results using digital neuropsychological and gait assessments within a prospective prediction model [14]. Compared with these approaches, our model integrates brief conventional paper‐and‐pencil cognitive assessments with routinely performed gait tests, providing a practical multidomain assessment strategy that can be completed within approximately 15–20 min without specialized equipment.

Several limitations should be acknowledged when interpreting the present findings. First, a major limitation of this study is the extremely small number of non‐responders in the surgical cohort, with only five non‐responders relative to the five predictors included in the integrated model. Although Firth penalized logistic regression and bootstrap internal validation were used to reduce small‐sample bias and estimate model optimism, these approaches cannot fully overcome the substantial uncertainty associated with the very small number of non‐responders. In addition, both the selection of the 24 h‐assessment time point and the determination of predictor cutoff values were data‐driven. The cutoff values were derived from the original surgical cohort and were subsequently fixed during bootstrap validation rather than being re‐estimated within each bootstrap resample. Therefore, the bootstrap procedure did not reproduce the entire model‐development process and may not fully account for optimism introduced by time‐point and cutoff selection. Consequently, the regression coefficients, cutoff values, specificity estimates, and model performance, including the optimism‐corrected AUC, should be interpreted cautiously. The integrated model should therefore be regarded as exploratory and hypothesis‐generating rather than as an established clinical prediction tool. Second, the model was internally validated using bootstrap resampling but has not undergone independent external validation. Consequently, its discrimination, calibration, and potential clinical utility require confirmation in larger prospective multicenter cohorts before routine implementation. Third, selection bias should be considered. In routine clinical practice at our institution, patients without a favorable response to CSF TT generally were not offered shunt surgery. As a result, postoperative outcomes were available only for patients who had already been selected for surgery, which likely enriched the cohort for responders and may have led to an overestimation of predictive performance. Therefore, the current model should be interpreted as applicable to patients selected for shunt surgery after standard preoperative evaluation rather than to all patients with possible iNPH. Finally, postoperative outcome was assessed three months after surgery using improvement of more than one point on the iNPH Grading Scale, providing a standardized measure of treatment response. However, follow‐up assessments were performed as part of routine clinical care and were not blinded to previous evaluations, and the relatively short follow‐up period precludes assessment of the long‐term stability of both surgical outcomes and model performance.

## 5 Conclusion

This study demonstrated that changes in cognitive processing speed and gait performance following CSF TT were associated with subsequent shunt responsiveness in patients with possible iNPH. Functional assessment performed 24 h after CSF TT provided the most informative evaluation of short‐term reversibility among the assessed time points. An integrated model combining cognitive processing speed and gait improvement showed better discrimination than individual measures. Although bootstrap internal validation yielded an optimism‐corrected AUC of 0.920, this estimate should be interpreted cautiously given the limited number of non‐responders and the data‐driven model‐development process. The present model should therefore be considered exploratory and hypothesis‐generating rather than an established clinical prediction tool. Larger prospective multicenter studies with longer follow‐up and independent external validation are warranted before routine clinical implementation.

## Author Contributions

Conceptualization: Ziang Geng, Junji Wei. Methodology: Ziang Geng, Zeyi Yang. Software: Rui Yin, Sishuai Sun. Data curation: Pengtao Li, Xiao Zhang, Yihao Chen, Jianbo Chang. Investigation; Xiaoyu Liu, Congcong Deng. Validation. Yue Wang, Ziyuan Fang. Formal analysis: Ziang Geng, Zeyi Yang. Supervision: Junji Wei, Ying Mao. Funding acquisition: Junji Wei. Visualization: Ziang Geng. Project administration; Junji Wei, Ying Mao. Resources: Junji Wei, Chenhui Mao, Jing Gao. Writing original draft: Ziang Geng, Zeyi Yang. Writing review and editing: Junji Wei, Ying Mao.

## Funding

This work was supported by CAMS Innovation Fund for Medical Sciences (Grant 2024‐I2M‐C&T‐B‐022), Science and Technology Major Project of Stroke Prevention and Treatment of the NHC‐Million Disability Reduction Initiative (Grant 2024PSPT0903102), and National High Level Hospital Clinical Research Funding (Grant 2025‐PUMCH‐A‐159).

## Ethics Statement

This study was approved by the Ethics Committee of Peking Union Medical College Hospital (2024‐I2M‐CT‐B‐022).

## Consent

Written informed consent was obtained from all patients or their legal representatives.

## Conflicts of Interest

The authors declare no conflicts of interest.

## Data Availability

Research data are not shared.
